# Clinical analysis of atlanto-occipital decompression in the management of chiari malformation with multi-segmental syringomyelia

**DOI:** 10.3389/fped.2024.1432706

**Published:** 2024-08-29

**Authors:** Yaning Sun, Jiangshun Fang, Shengjuan Wang, Jimei Luan, Na Wang, Lige Lv, Chaojun Xin, Pengyuan Luo, Yanke Yue, Zhenghai Cheng, Zhiguo Yang, Liuyin Chen

**Affiliations:** Department of Neurosurgery, Hebei Children’s Hospital, Shijiazhuang, Hebei, China

**Keywords:** atlanto-occipital decompression, chiari malformation type I, multi-segmental beaded syringomyelia, pediatric, clinical analysis

## Abstract

**Background:**

Chiari malformation type I with syringomyelia (CM-I-S) is a very common disease in neurosurgery. There are also various surgical methods. But it is controversial. There are few reports about children, especially the treatment of multi-segmental beaded syringomyelia. The purpose of this study was to explore the clinical effects of atlanto-occipital decompression (AOD) in the management of Chiari malformation type I (CM-I) with multi-segmental beaded syringomyelia (MSBS) in pediatric patients.

**Methods:**

This retrospective study were pediatric patients with CM-I combined with MSBS who were treated in our hospital from January 2015 to December 2023. The patients who received the AOD treatment were screened according to inclusion and exclusion criteria. Outcomes were assessed by comparison of pre- and postoperative clinical, Chicago Chiari Outcome Scale (CCOS), the diameter and volume of the syringomyelia, morphological parameters of posterior cranial fossa (cervical spinal cord angle of medulla oblongata, CSC-MO) and complications in the enrolled children.

**Results:**

This study ultimately included 21 eligible pediatric patients with CM-I and MSBS. All the patients successfully completed the operation, which consists of atlanto-occipital decompression, partial resection of the posterior arch of the atlas, electrocoagulation of the cerebellar tonsil, pseudomembrane resection of the central canal orifice (latch) of the spinal cord, and artificial dura mater expansion repair. No death, no relapses, no serious neurological dysfunction and other complications. At the last follow-up, the clinical symptoms of all patients basically disappeared. The results of magnetic resonance imaging (MRI) showed that the average preoperative cerebellar tonsillar hernia was 12.4 ± 4.6 mm, and the postoperative were all above the foramen magnum. The average preoperative syringomyelia volume was 11.7 ± 3.7 cm^2^, and the syringomyelia disappeared or significantly shrunk after operation, with the volume unable to be accurately measured. The median preoperative CSC-MO was 132.5°, and the median postoperative CSC-MO was 150°, with a significant difference. The median preoperative pain and non-pain score of CCOS was 4 and 3 respectively. The symptoms disappeared after operation, and the score was 4. Only 5 children had cerebrospinal fluid leakage.

**Conclusion:**

The AOD is safe and effective in CM-I with MSBS in pediatric patients. Pseudomembrane resection of the central canal orifice (latch) of the spinal cord is crucial for the treatment of syringomyelia.

## Introduction

The common cause of Chiari malformation (CM) is structural abnormalities at the craniovertebral junction caused by developmental defects in the central nervous system during embryonic development, in which the abnormal inferior part of the cerebellar tonsil extends in a spiked shape and herniates downward into the cervical vertebral canal below the foramen magnum (1). Patients with CM often have different degrees of syringomyelia (2). Headache is the most common clinical symptom in CM, accounting for 40%–60% (3–6). Some patients will also have pain at the occipital-cervical junction. Especially when Valsalva maneuvers, it can aggravate the pain in occipital and upper neck (7, 8). With the aggravation of the degree of lower hernia, the clinical symptoms will gradually worsen, resulting in numbness of limbs, ataxia, sensory separation and even hydrocephalus (8). In pediatric patients, some young children cannot accurately describe uncomfortable symptoms due to their immature language skills, making it easier for pediatric CM to be missed or misdiagnosed. However, pain can cause children to become irritable and frequently cry. Therefore, all patients who cannot provide detailed symptoms should be given special attention to avoid missed or misdiagnosed cases.

There are four main types of CM, with type I being the most common in pediatric patients. It refers to a congenital malformation in which the tonsil of the cerebellum (≤14 years old) protrudes more than 5 mm below the foramen magnum. Additionally, 25% of CM-I patients also have syringomyelia (9, 10). The current mechanism of syringomyelia is not clear, and it is considered to be related to changes in cerebrospinal fluid (CSF) dynamics (11). It is generally believed that surgical treatment is the first choice for patients with CM-I-S, and the earlier they receive treatment, the fewer clinical sequelae and better results they will have (12, 13). Over time, some patients with CM-I-S have gradually worsened, causing continuous compression of the spinal cord and even the possibility of paralysis. At the same time, due to missed diagnosis or other reasons, some children have already developed multilevel syringomyelia at the time of diagnosis, accompanied by neurological symptoms. Therefore, surgical treatment should be performed promptly after the discovery of CM-I-S.

Multiple segmental syringomyelia refers to a syringomyelia involving more than two parts of the cervical, thoracic, lumbar, and sacral regions. Children with CM-I with MSBS should be given special attention. Because the length and diameter of MSBS are larger than other types of syringomyelia, and the neurological symptoms are more pronounced. Correct treatment can maximize the improvement of the patient's prognosis and reduce the incidence of neurological sequelae. The correct treatment can maximize the improvement of the prognosis of children and reduce the incidence of neurological sequelae. Currently, most of the references available for reading are based on adult patients, excluding children. There are few reports on the clinical manifestations, surgical methods, prognosis and complications of CM-I with MSBS in pediatric patients. Common surgical procedures for adults include posterior fossa bone decompression, dural enlargement repair, and cerebellar tonsillectomy. Patients with severe syringomyelia undergo central canal opening release, syringomyelia incision drainage, or syringomyelia shunt surgery. However, there is currently no unified treatment standard, and treatment methods also have certain controversies.

This study conducted a retrospective analysis of the clinical data of CM-I with MSBS patients who underwent AOD in our hospital. Objective evaluation was conducted by comparing the clinical symptoms and imaging findings before and after operation, analyzing various data and indicators, summarizing treatment experience, and evaluating the safety and effectiveness of this surgical approach.

## Materials and methods

### General information

This study retrospectively analyzed pediatric patients with CM-I combined with MSBS who received AOD treatment in our hospital from January 2015 to December 2023. The institutional review board approved the study, and all parents of the young child patients provided informed consent for study inclusion. (Ethics Number: 20240916)

Inclusion criteria: (1) age ≤14 years old. (2) MRI showed that the cerebellar tonsillar hernia exceeded the foramen magnum by more than 5 mm. (3) More than two segments of syringomyelia: cervical-thoracic syringomyelia, cervical-thoracic-lumbar syringomyelia, or total syringomyelia. (4) Syringomyelia presents as a beaded appearance. (5) Parents sign informed consent for operation and postoperative follow-up. (6) No other medical history, and can tolerate surgery.

Exclusion criteria: (1) Children with poor perioperative compliance. (2) CM-I without syringomyelia. (3) Single-segment syringomyelia. (4) Severe scoliosis (5) Genetic metabolic diseases. (6) Unable to tolerate surgery. (7) No informed consent provided by the legal representative of the pediatric patient.

### Clinical evaluation

The clinical symptoms and the degree of cerebellar tonsillar hernia were compared before operation and at the last follow-up. Chicago Chiari Outcome Scale (CCOS) was used to evaluate the surgical effect. The diameter, radius and volume of syringomyelia were recorded. The morphological parameters of posterior cranial fossa, that is, the angle of medulla oblongata, were compared. Postoperative complications (headache, wound infection, cerebrospinal fluid leakage and intracranial infection) were recorded.

### Operation procedures

**Figure 1:**
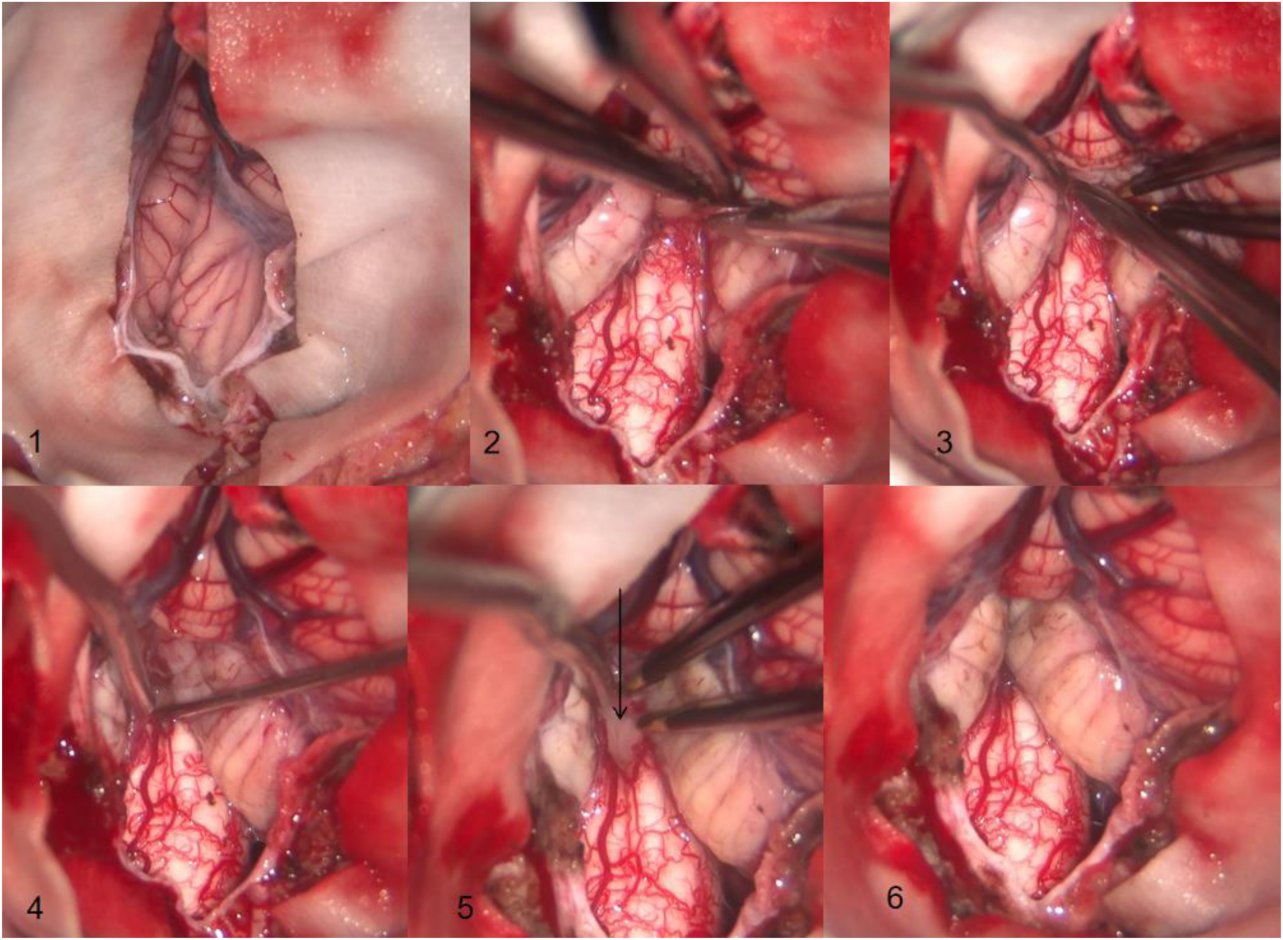
**(1)** The tonsil of the cerebellum can be seen descending into the foramen magnum and adhering to the surrounding tissues **(2)** the soft membrane of cerebellar tonsil is coagulated by bipolar electrocoagulation, and the arachnoid membrane adhered to medulla oblongata is separated at the same time. **(3)** The tonsil of the cerebellum is separated from the arachnoid membrane adhering around the magendie foramen. **(4)** The magendie foramen is opened and the central canal of the spinal cord is explored. **(5)** The membrane at the central canal of the spinal cord can be seen at the arrow. **(6)** The adhesion of the tonsil of the cerebellum to the medulla oblongata, magendie foramen, and other tissues has been completely loosened, and the tonsil of the cerebellum is clearly located above the foramen magnum through electrocoagulation rather than resection.

After satisfactory anesthesia, the patient is placed in a prone position with the neck flexed forward and a head rest used to secure the head. A posterior midline incision is made between the 1 cm below the external occipital protuberance and the second spinous process of the neck, and the skin and subcutaneous tissues are sequentially incised along the white line. The bilateral posterior occipital muscle tissues are separated, exposing the area between the occipital bone and the second spinous process of the neck. Remove the occipital squamous bone flap and the posterior edge of the foramen magnum (the size of the bone flap is approximately 4 cm × 4 cm) in sequence. Use a rongeur to bite off part of the posterior arch of the atlas (approximately 1 cm wide). Remove the thickened atlanto-occipital fascia. Y-shaped incision was made into the dura mater, and the dural incision extended downward to the level of the lower edge of the atlas. It can be seen that the bilateral cerebellar tonsils exceeded the lower edge of the foramen magnum by 0.5 cm, compressing the cervical spinal cord. Using bipolar low-frequency electrocoagulation under a microscope, cauterize the soft membrane of the cerebellar tonsil to shrink and move up to a level 5 mm above the foramen magnum, relieving the compression on the medulla oblongata and cervical spinal cord. Explore the fourth ventricle, if the Magendie foramen is adherent or closed, the adherent arachnoid membrane should be separated to promote the circulation of CSF. A membrane (false membrane) can be seen at the central canal of the spinal cord at the lower end of the fourth ventricle. After the sharp knife slice cuts the false membrane, a nerve hook is used to explore the interior of the central canal opening, and a syringe is used to flush open the closed segment within the central canal. The dural mater is expanded and repaired using a 4 cm × 4 cm bio-type dural patch. After checking for no cerebrospinal fluid leakage and no active bleeding, the surgical incision is sutured layer by layer (Figure 1).

### Postoperative care

Postoperatively, the cephalic CT was performed to check for bleeding. To avoid postoperative cerebrospinal fluid leakage, the surgical incision is pressure-wrapped with bandages. After 8–10 days, the sutures are removed based on the healing of the surgical incision.

### Statistical analysis

SPSS 26.0 (IBM, Armonk, New York, USA) software was used to statistically analyze the preoperative and postoperative data of patients. Data collected at the last visit were used for comparisons with the preoperative baseline parameters. The measurement data that conformed to the normal distribution were expressed as mean ± standard deviation. Using quartiles and non-parametric methods to calculate data that do not conform to a normal distribution. The paired measurement data *t*-test was used for the comparison of pre- and post-operative scores, and *P* < 0.05 was considered statistically significant.

## Results

### General information

Twenty-eight patients with CM-I with MSBS treated in the Department of Neurosurgery between January 2015 and December 2023 were screened for study eligibility. After seven ineligible patients were excluded, the total study cohort comprised 21 eligible pediatric patients with CM-I with MSBS who received atlanto-occipital decompression, partial resection of the posterior arch of the atlas, electrocoagulation of the cerebellar tonsil, pseudomembrane resection of the central canal orifice (latch) of the spinal cord, and artificial dura mater expansion repair. Among them, there were 12 males and 9 females. The age ranged from 1 to 13 years (average 7.4 ± 3.29 years). All included patients completed follow-up. The median hospitalization time was 14 days. The median follow-up time was 2.75 years. No death, no relapses, no serious neurological dysfunction and other severe complications.

### Clinical symptoms

The clinical symptoms of the 21 patients were different. Six patients had dizziness symptoms. One patient had neurological symptoms, namely weakness of both lower limbs. One patient had occipital-cervical pain at the same time. One patient had MRI results suggesting hydrocephalus. Seven children were admitted due to abnormalities in the nervous system. Five children presented with weakness in both lower limbs, including one child with weakness in both lower limbs accompanied by weakness in the upper limbs, and one child who was prone to choking when drinking water. Two children complained of occipital-cervical pain. Two children went to see a doctor because of convulsion, and the results of head MRI suggested CM-I, but one of them developed hydrocephalus. CM-I was found in a child with scoliosis. One child went to see a doctor because of the enlarged head circumference. The MRI showed hydrocephalus with CM-I-S. One child was admitted to the hospital after head trauma, and cranial MRI showed CM-I with a 0.5 cm herniation of the cerebellar tonsil. There were no obvious symptoms. One year later, a follow-up examination showed that the degree of herniation had increased to 1.0 cm, and a syringomyelia had appeared.

### Cerebellar tonsil

The preoperative tonsillar herniation of pediatric patients was about 12.4 ± 4.6 mm, and the tonsils were all above the foramen magnum at the last follow-up, with no recurrence. The difference was statistically significant (*P* < 0.05).

### Syringomyelia

**Figure 2:**
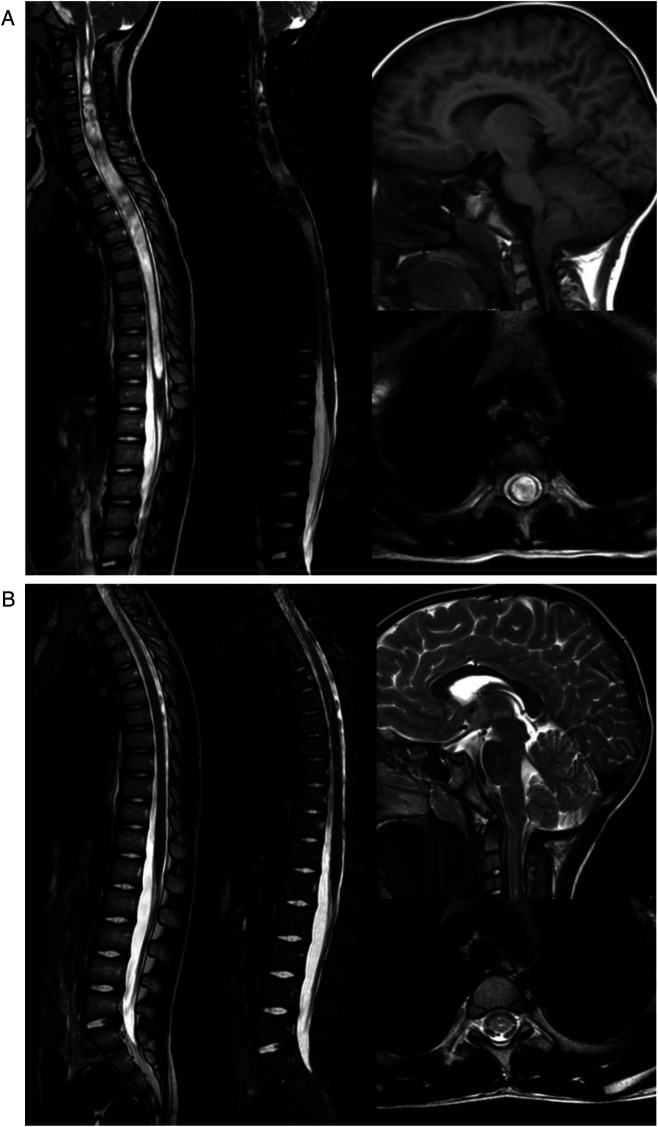
**(A)** This is a 4-year-old boy who presented with dizziness and occipital-cervical pain. The MRI results of the cephalic and spinal cord showed that the medulla oblongata and pons were compressed and deformed, the foramen magnum was crowded, and the inferior margin of the cerebellar tonsil exceeded the level of the foramen magnum, about 1.1 cm. Multiple beaded-like abnormal signals were visible in the spinal cord. The lesion extended up to the clivus and down to the inferior margin of the 11th thoracic vertebra. The diameter of the syringomyelia was about 1.0 cm, the length of the syringomyelia was 22.55 cm, and the volume of the syringomyelia was 17.7 cm^3^. The CSC-MO was 102°. **(B)** This was reviewed 2 years after operation. The MRI results of cephalic and spinal cord showed that the medulla oblongata and pons were normal, the cerebellar space was clear, and the lower edge of cerebellar tonsil was located above the foramen magnum. The multiple beaded signals in the spinal cord have disappeared, and the compression of the spinal cord parenchyma has decreased compared to pre-operative findings. The length and diameter of the syringomyelia after surgery cannot be measured. The CSC-MO was 155°.

In this study, syringomyelia is compared to a cylinder, and the volume of syringomyelia is roughly calculated by using the formula for calculating the volume of cylinder, and the degree of syringomyelia before and after operation is compared. The volume of the cylinder is calculated as volume=π×hr2.π=3.14, where r represents the radius of the maximum cavity in the axial position of the syringomyelia. h is the height of the cylinder, which is the length of the syringomyelia. Although the syringomyelia is not a regular cylinder, the volume of the syringomyelia in all children before and after surgery is calculated using this formula, and the results can be considered objective and fair. At the last follow-up, MRI of the entire spinal cord showed that the syringomyelia in all children had significantly improved compared to pre-operative findings, with significant changes in the width and length of the syringomyelia compared to pre-operative findings. The average diameter of the syringomyelia before surgery was 0.9 ± 0.1 cm, the average length of the syringomyelia was 18.1 ± 2.1 cm, and the average volume was 11.7 ± 3.7 cm^3^. The diameter, length, and volume of the syringomyelia after surgery could not be accurately calculated. The difference was statistically significant (*P* < 0.05). The median preoperative CSC-MO was 132.5°, and the median postoperative CSC-MO was 150°. Compared with preoperative, there was significant difference, which was statistically significant (*P* < 0.000) (Figure 2).

### Complications

Five patients developed cerebrospinal fluid leakage after surgery. After puncturing the cerebrospinal fluid and suctioning it, sterile dressing was applied and pressure dressing was applied. The symptoms of cerebrospinal fluid leakage disappeared. Absorbable sutures was not absorbed in the surgical incision in one child, and the incision was infected locally, which healed after dressing change.

The median preoperative pain and non-pain score of CCOS was 4 and 3 respectively. Both the pain and non-pain symptoms disappeared after surgery, and the scores were 4 points. At the last follow-up, all children reported no discomfort in their upper and lower limbs, and all entered school normally. The results of CCOS score after operation confirmed that the condition recovered well (Table 1).

**Table 1:** The detailed condition of all patients includes: general information, clinical symptoms, pain and non pain points, length of cerebellar tonsillar hernia, position of syringomyelia, diameter, length and volume, as well as preoperative and postoperative cervical spinal cord angle of medulla oblongata.

Patient	Age year	Sex	Symptom	C-P	C-NP	L-CTH mm	S-S	D-S cm	S cm	V-Scm3	Pre-CSC-MO (°)	Post-CSC-MO (°)
1	8	F	Upper limb shaking, lower weakness	4	3	19	C-T-L	1.1	20.55	19.5	128	150
2	12	F	Hypoesthe-sia of left upper limb	4	3	16	C-T	1	16.28	12.8	130	148
3	4	F	Headache	2	4	17	C-T	0.8	17.78	8.9	135	160
4	9	F	Convulsion	4	2	17	C-T	0.7	16.25	6.3	128	152
5	9	F	Weakness, choking from drinking water	4	3	23	C-T	1	15.22	12	119	145
6	4	F	Seizure, hydroceph-alus	4	3	5	C-T	0.9	14.78	9.4	138	155
7	7	F	Dizziness with hydroceph-alus	4	3	10	C-T	0.8	16.37	8.2	140	167
8	3	F	Found after injury	4	4	10	C-T	1	15.84	12.4	135	149
9	9	F	Headache	2	4	7	C-T	0.9	19.85	12.6	133	145
10	1.5	M	Hydroceph-alus	4	2	7.5	C-T	0.9	18.36	11.7	140	158
11	13	M	Left finger movement limitation	4	2	12	C-T	1	19.5	15.3	130	142
12	10	M	Tethered spinal cord	4	2	7.1	C-T	1	18	14.1	128	163
13	5	M	Scoliosis, dizziness	4	2	12	C-T	1.1	17.96	17.1	132.5	148.5
14	4	M	Head and neck pain, dizziness	3	3	11	C-T-L	1	22.55	17.7	102	155
15	13	M	Dizziness, weakness	4	2	13	C-T	1	19.5	15.3	135	165
16	7	M	Dizziness	4	2	18	C-T-L	0.7	21.85	8.4	125	145
17	4	M	Weakness of lower limbs	4	3	14	C-T	0.8	16.74	8.4	133	144
18	6	M	Dizziness	4	2	12	C-T	0.8	17.73	8.9	140	150
19	9	M	Weakness	4	2	10	C-T	0.9	16.88	10.7	134	146
20	8	M	Dizziness	4	3	9	C-T	0.7	19.15	7.3	129	150
21	10	M	Weakness	4	2	11	C-T	0.8	18.85	9.5	128	138

Ccos pain: C-P (points) Ccos non-pain: C-NP (points).

Length of cerebellar tonsillar hernia: L-CTH (mm).

Diameter of syringomyelia: D-S (cm).

Length of syringomyelia: L-S (cm) The volume of syringomyelia: V-S (cm^3^).

The site of syringomyelia: S-S.

Cervical and thoracic segments: C-T Cervical, thoracic and lumbar segments: C-T-L.

Cervical spinal cord angle of medulla oblongata: CSC-MO (°).

## Discussion

CM-I is a disease whose main imaging feature is that the inferior margin of cerebellar tonsil exceeds the level of foramen magnum. The main pathogenesis is thought to be caused by occipital dysplasia caused by paraxial dysplasia of embryonic axial lobe (1, 2, 14). The hypoplasia of occipital bone will lead to the thickening of occipital scale bone, which will lead to the reduction of posterior cranial fossa volume. However, the brain tissue in the posterior cranial fossa develops normally, which leads to overcrowding of brain tissue, and then leads to cerebellar tonsillar hernia into the foramen magnum. Liu et al. (15) measured the bony landmarks of the posterior cranial fossa in 38 patients with CM-I and compared them with those of 30 healthy volunteers. The results showed that the length of the posterior cranial fossa clivus, suprasellar length, posterior cranial fossa sagittal diameter, and clivus inclination angle in patients with CM-I were all smaller than those in normal people. Hofkes et al. (16) found through research that the volume of the cerebellum/cranial fossa was significantly smaller than the normal anatomical relationship. This study also supports the hypothesis of developmental disorders in the posterior cranial fossa after CM-I, in which the brain tissue develops normally but the bony structures of the posterior cranial fossa become smaller. At the same time, CM-I can also lead to CSF circulation disorders and cause a series of neurological symptoms in children. When the flow of CSF is blocked, the impact of pulsation in the choroid plexus of the ventricles is transmitted downward, causing the central canal of the spinal cord to expand, forming a syringomyelia (11, 13, 17). Therefore, no matter what surgical method is chosen, the purpose of treatment is to enlarge the space of the posterior cranial fossa, reconstruct the foramen magnum area, restore the normal brain tissue volume, and restore the normal CSF circulation, thus alleviating the clinical symptoms of children.

Posterior cranial fossa decompression, cerebellar tonsillectomy and duraplasty are the most common surgical methods for CM-I (18). Although these surgical methods expand the volume of the posterior cranial fossa to a certain extent, they have certain disadvantages, namely, excessive or incomplete decompression, which increases the risk of secondary cerebellar ataxia and recurrence of CM-I (19). During posterior cranial fossa decompression, the dura mater is opened but not sutured, which results in the loss of normal dural anatomical structures in the cerebellar tissue, destroying the anatomical protective structure of the external scaffold, leading to the emergence of related potential complications (20–22). Some surgeries use large bone windows (6 × 6 cm) to expand the volume of the posterior cranial fossa, which can provide sufficient decompression in the short term, but the cerebellum loses its bony support, which can also easily lead to recurrence of cerebellar ptosis and deformity (23). Some scholars have attempted small bone window decompression to prevent cerebellar ptosis, but the bone flap is too small, which can lead to incomplete decompression (24) Although dural plasty in the posterior cranial fossa has been performed on the basis of open decompression in the posterior cranial fossa, it has not fundamentally resolved the compression of the medulla oblongata by the herniated tonsil of the cerebellum. Some patients with CM-I-S are accompanied by symptoms of atlantoaxial dislocation, for whom occipito-cervical fusion and internal fixation are also performed during decompression surgery (25). The above bony and membranous decompression procedures for the posterior cranial fossa have different clinical effects on alleviating the compression symptoms caused by CM-I, but they do not significantly improve the multistage beaded-like changes in syringomyelia.

In view of the advantages and disadvantages of the above surgical methods, this study has improved the above surgical methods. All pediatrics were treated with AOD, which consists of occipital decompression, partial resection of the posterior arch of the atlas, electrocoagulation of the cerebellar tonsil, pseudomembrane resection of the central canal orifice (latch) of the spinal cord, and artificial dura mater expansion repair. The size of the bone window in this study was 4 × 4 cm, which was moderate in size, avoiding the possibility of secondary drooping of the cerebellum caused by an overly large bone window, while also avoiding the drawbacks of insufficient decompression caused by an overly small bone window. After removing the squamous part of the occipital bone, part of the posterior arch of the atlas was bitten off (approximately 1 cm wide). The thickened atlanto-occipital fascia was removed. This approach also aims to increase the volume of the posterior cranial fossa, relieve pressure on neural tissue, and improve blood and cerebrospinal fluid circulation (26). Will removing part of the posterior arch of the atlas affect the stability of the craniovertebral junction? The answer is no. Because the stability of the atlantoaxial joint mainly depends on the apical ligament and the cruciate ligament. The surgery in this study does not have a significant impact on the stability of the atlantoaxial joint (27). However, for safety reasons, we advise parents to carefully monitor their children after surgery to avoid excessive neck extension and flexion activities. Through follow-up after surgery, all children had good stability at the craniovertebral junction, and no serious complications occurred.

The cerebellum is a very important organ in the human body, mainly controlling the balance, language, and emotional functions of the body (28, 29). For example, resection of medulloblastoma in the cerebellar region may lead to mutism in patients. As an important structure of the cerebellum, the function of the tonsil of the cerebellum is not yet clear. Is there any effect on the human body after the tonsil of the cerebellum is removed? The answer is unknown. Related studies have followed up on patients after tonsillectomy and found that some patients have subtle eye movement changes that they cannot detect themselves (30). The conventional surgical approach is to remove part of the herniated cerebellar tonsil to relieve the compression on the medulla oblongata. Some literature also suggests that only by removing the herniated cerebellar tonsil can the CSF pathway in the fourth ventricle become more unobstructed. The circulation of CSF is improved, and the clinical symptoms of hydrocephalus and syringomyelia can be improved to a certain extent (1, 2, 12, 18). However, removing the tonsil of the cerebellum will prolong the operation time and increase the amount of intraoperative bleeding. At the same time, it may cause damage to the medulla oblongata and cervical cord. In this study, we used low-frequency bipolar electrocoagulation to constrict the arachnoid and pia mater around the tonsil of the cerebellum, causing the tip of the tonsil of the cerebellum to contract upward and outward, relieving the pressure on the medulla oblongata and upper cervical cord, releasing the adhesion of the median foramen, ensuring the permanent opening of the magendie, and restoring the circulation of CSF. Although using low-frequency bipolar electrocoagulation to constrict the arachnoid of the cerebellum will also cause some damage to the cerebellum, compared with resection, electrocoagulation causes less damage to the tonsil of the cerebellum. Among the children who were followed up after surgery, the longest follow-up time was 8.7 years, and there were no relapses.

Iwasaki et al. (31) believed that the sagittal diameter of syringomyelia is ≥70% of the spinal cord diameter, and the local spinal cord is obviously thickened, so the tension syringomyelia should be treated by shunt operation. Fujii et al. (32) believed that shunt surgery can be performed when the diameter of the syringomyelia is greater than 35%. Common shunt surgeries mainly include syringomyelia-subarachnoid shunt, syringomyelia-pleural shunt, and syringomyelia-abdominal shunt. However, shunt surgery has certain risks. There are complications such as tube breakage and blockage. And there are few relevant case reports in pediatric patients. According to literature reports, the pseudomembrane of the central canal orifice (latch) of the spinal cord is one of the main causes of CMS, and opening the pseudomembrane of the latch is the fundamental method for treating syringomyelia (20) This surgical approach has a lower risk compared to shunt surgery. Zhang Yuqi et al. (20) performed a central canal opening release procedure on 15 patients with CM-I-SM, and found that in patients with mild syringomyelia, the syrinx completely disappeared at the last follow-up. In patients with severe syringomyelia, the syrinx can be significantly reduced after surgery, but it cannot completely disappear. As time goes on, the syrinx may continue to shrink and even disappear. This study reached the same conclusion as theirs. At the last follow-up after, most of the patients’ syringomyelia significantly decreased, and even the volume of the syrinx could not be measured. Some patients’ syringomyelia disappeared.

In the treatment of patients with CM-I-S, we should strengthen our understanding of the anatomy of the fourth ventricle and the opening of the central canal of the spinal cord. Because syringomyelia may be related to the abnormal circulation of CSF (13). The fourth ventricle is the last ventricle of CSF circulation. It connects the aqueduct of the midbrain above and the central canal of the spinal cord below. It communicates with the subarachnoid space through magendie and lateral foramen. If the descending tonsil of the cerebellum adheres to the median foramen, or the opening of the central canal of the spinal cord is not smooth, it may be the cause of syringomyelia. Therefore, we should explore the magendie foramen of the fourth ventricle and the opening of the central canal of the spinal cord during surgery. I believe there are scholars who believe that the operation at the opening of the central canal of the spinal cord poses significant risks, including potential damage to the medulla oblongata. For patients with syringomyelia, the fluid in the syrinx oppresses the spinal cord over time, leading to thinning of the spinal cord cortex. This can also be seen as abnormally thinning of the spinal cord on an MRI scan. Additionally, the surgery is performed under a microscope, and we ensure that the opening of the central canal is a vascular-free membranous structure before proceeding. Furthermore, the operator is an experienced chief physician.

There is also some controversy regarding the treatment of CM-I-SM with hydrocephalus. Should the focus be on treating hydrocephalus first? Can CM-I-SM be relieved after the hydrocephalus is alleviated? Or should the focus be on treating CM-I-SM first through occipital decompression? Can the ventricles return to normal after the relief of CM-I-SM? Vernet et al. (33) believed that ventriculoperitoneal shunt was the main method for treating CM-I with hydrocephalus. Hayhurst et al. (34) believed that the effect of endoscopic third ventriculostomy was better. They analyzed and studied 15 children with CM-I with or without syringomyelia. After surgery, 5 of the 6 patients with syringomyelia showed improvement. Six patients underwent decompression of foramen magnum again because of the persistent symptoms of CM-I or syringomyelia. However, Sharma et al. (35) believed that the reason of cerebellar tonsillar hernia leads to obstruction of the outflow tract of the fourth ventricle and secondary hydrocephalus. Therefore, first performing decompressive surgery on the posterior cranial fossa, the symptoms of obstructive hydrocephalus will also improve. In this study, there were 3 patients with symptoms of hydrocephalus in CM-I-SM, all of whom were treated with atlanto-occipital decompression surgery. Follow-up analysis after surgery showed that the symptoms of hydrocephalus in all 3 patients improved compared to before surgery.

The CSC-MO was first proposed by Bundschuh et al. (36) in 1988 and is considered to be the most effective assessment method for evaluating the degree of compression and surgical reduction of the medulla oblongata and cervical spinal cord (37). Bundschuh et al. (36) reported a normal CSC-MO of 155.2° on average, while Abumi et al. (38) reported a normal range of 154°–179°. Tian et al. (39) measured the CSC-MO of 117 Chinese people through MRI, and the normal range was 130.38°–168.75°, with an average of 154.17°. In this study, the median angle of the CSC-MO was 132.5° before surgery and 150° after surgery, with significant differences and statistical significance, indicating that the compression of the CSC-MO had significantly improved. The post-operative CSC-MO was similar to previous literature research results. Puschak et al. (40) believed that when the CSC-MO was less than 135°, it indicated that the brain stem was significantly compressed, and the patient had neurological damage symptoms, and the atlantoaxial fusion surgery should be performed. However, in this study, considering the young age of the patient and the mild neurological symptoms, all the patients did not undergo fusion surgery. After surgery, the neurological symptoms disappeared and the CSC-MO basically returned to normal.

According to literature reports, headache is the most common clinical manifestation in pediatric patients with CM-I, with a high incidence of up to 80% (5, 6). In this study, a total of 21 pediatric patients were included, and only 3 patients experienced occipital neck pain, accounting for approximately 14.3%. The highest proportion of neurological symptoms occurred in this study, with a total of 8 patients (38.1%) experiencing varying degrees of weakness in both lower limbs or upper limb dysfunction. Dizziness was the second symptom, with 6 cases experiencing dizziness. Due to increased head circumference, CM-I-S was detected during the examination, and cases of hydrocephalus and occipital-cervical pain occurred at the same rate, accounting for 14.3% of the total. In addition, there were also patients who were admitted for convulsions. It is worth discussing that the clinical symptoms in this study are inconsistent with the most commonly reported clinical symptoms in previous literature. There are several reasons for this. Firstly, the inclusion criteria in this study were CM-I-MSBS, with significant compression of the spinal cord parenchyma, so the occurrence of neurological symptoms in such patients are more pronounced. Secondly, children are largely guided by parents to identify and judge pain symptoms, which will also affect children's subjective description of pain. Thirdly, the pediatric patients may not have a clear description of the clinical symptoms. However, the weakness of both lower limbs or upper limb dysfunction can be observed by the parents. Finally, there were a small number of cases in this study, which may have resulted in some errors.

This study suffered from several limitations. First, the retrospective design might have impeded the accuracy and precise in data collection. Second, due to the limited use in our institution, only 21 eligible patients were included for data analysis, making the comparison not definitely conclusive. Third, the single-center design would have lowered the generalizability of our results to other settings.

## Conclusion

The AOD is safe and effective in CM-I with MSBS in pediatric patients. Pseudomembrane resection of the central canal orifice (latch) of the spinal cord is crucial for the treatment of syringomyelia. However, it should be analyzed according to the specific condition of the patient and individualized treatment should be carried out.

## Data Availability

The original contributions presented in the study are included in the article/Supplementary Material, further inquiries can be directed to the corresponding author.
